# Temperature Profile in Rubber Injection Molding: Application of a Recently Developed Testing Method to Improve the Process Simulation and Calculation of Curing Kinetics

**DOI:** 10.3390/polym13030380

**Published:** 2021-01-26

**Authors:** Martin Traintinger, Roman Christopher Kerschbaumer, Bernhard Lechner, Walter Friesenbichler, Thomas Lucyshyn

**Affiliations:** 1 Polymer Competence Center Leoben GmbH, Roseggerstrasse 12, 8700 Leoben, Austria; Roman.Kerschbaumer@pccl.at (R.C.K.); Bernhard.Lechner@pccl.at (B.L.); 2 Department of Polymer Engineering and Science, Chair of Injection Moulding of Polymers, Montanuniversitaet Leoben, Otto Gloeckel-Strasse 2, 8700 Leoben, Austria; Walter.Friesenbichler@unileoben.ac.at; 3 Department of Polymer Engineering and Science, Chair of Polymer Processing, Montanuniversitaet Leoben, Otto Gloeckel-Strasse 2, 8700 Leoben, Austria; Thomas.Lucyshyn@unileoben.ac.at

**Keywords:** injection molding, rubber compound, simulation, mass temperature prior to injection, degree of cure

## Abstract

Injection molding of rubber compounds is an easily conducted yet sophisticated method for rubber processing. Simulation software is used to examine the optimal process conditions, identify failure scenarios, and save resources. Due to the complexity of the entire process, various aspects have to be considered in the numerical approach. This contribution focused on a comparison of process simulations with various definitions of the material’s inlet temperature, ranging from a stepwise increase, but constant temperature, to an exact axial mass temperature profile prior to injection. The latter was obtained with a specially designed, unique test stand consisting of a plasticizing cylinder equipped with pressure sensors, a throttle valve for pressure adjustments, and a measurement bar with thermocouples for the determination of the actual state of the mass temperature. For the verification of the theoretical calculations, practical experiments were conducted on a rubber injection molding machine equipped with the mold used in the simulation. The moldings, obtained at different vulcanization time, were characterized mechanically and the results were normalized to a relative degree of cure in order to enable comparison of the real process and the simulation. Considering the actual state of the mass temperature, the simulation showed an excellent correlation of the measured and calculated mass temperatures in the cold runner. Additionally, the relative degree of cure was closer to reality when the mass temperature profile after dosing was applied in the simulation.

## 1. Introduction

Injection molding of rubber has become a very powerful manufacturing method to obtain dozens of products like sealings, housings, dampings, and other technically sophisticated parts in a rather short processing times compared to other techniques, such as compression or transfer molding. However, the rubber injection molding (IM) process is quite sensitive, and its success depends on numerous aspects related to the rubber compound and the process settings. For this reason, many people put a lot of effort into developing various improvements for simulation software, such as SIGMASOFT^®^ from SIGMA Engineering GmbH (Aachen, Germany), to visualize the entire process [1,2,3,4]. One of the most challenging aspects is the flow behavior of rubber compounds, which is different to that of their thermoplastic relatives. While thermoplastics are usually injected around the melt temperature of the polymer to obtain the required flow of the material through a hot runner system [5], rubber compounds only admit temperatures between 70 –110 °C [6], since a premature start of the curative reaction in the formulations must be strictly avoided. In this phase of the process, rubber compounds exhibit a higher flow resistance, indicating a higher viscosity of the formulation compared to that of their thermoplastic counterparts. This illustrates the importance of understanding the complex characteristics of rubber compounds and the physical correlations between material flow and viscosity.

Both thermoplastic and elastomer material groups with pure molten, unfilled polymers, and a high molecular weight typically show a Newtonian plateau at small deformation rates. The plateau corresponds to the linear viscoelastic region of the polymer, which is shifted to even smaller deformation rates with increasing presence of fillers [7,8]. This behavior is similar to that of the complex composition of rubber compounds, which generally contain one or more polymers on either a synthetic or natural base; carbon black or silica-based materials as reinforcing fillers; several additives with different functions, such as increasing the resistance to aging; and a curative system, usually of sulfuric or peroxidic origin, which provides the formation of chemically cross-linked parts [9]. In view of the wide variety of possible rubber formulations, physical interactions of the constituents play an important role in material flow. Branched polymers with high molecular weights, for example, show a higher flow resistance than do polymers with short chains as a result of a higher degree of entanglement. Styrene butadiene rubber (SBR) is a particularly good example of this, as different macrostructures can be obtained depending on the polymerization method used. Cold emulsion polymerization at 5 °C leads to a branched but comparatively linear polymer, whereas the same polymerization in tempered media (between 40–50 °C) tends to form branched chains. For this reason, plasticizers are added to reduce the viscosity of the compound [9]. Another physical effect influencing the material flow is the reinforcement by filler particles, often referred to as the “Payne effect” [10]. Froehlich et al. [11] ascribed the reason to the primary particle size, the specific surface area and its degree of irregularity, and the surface activity of the filler, thus leading to more or less interaction of the fillers with the other contents of the rubber compound.

Apart from the morphology of a rubber compound, the viscosity of the material is strongly dependent on the bulk temperature. In rubber processing, however, this temperature is not only the result of externally applied heat, but also of heat dissipation, which occurs due to shear and elongational heating and viscoelastic effects during the flow through a die or a runner system. Consequently, the mass temperature of the rubber during processing is less of a homogeneous field and more of a non-uniform and time-varying field leading to a unique profile that affects the whole cycle [4]. Perko et al. [12,13] focused their work on targeted exploitation of heat dissipation with the aim of reducing the cycle time in rubber injection molding. Their approach is that the required time for vulcanization is reduced if the bulk temperature is increased simultaneously during dosing and/or filling. Therefore, they developed a new viscous model by means of the energy conservation equation for the prediction of temperature changes in rubber compounds flowing through conical dies. For each section of a discretized die, the calculations not only consider the shear heating effect, but also elongational viscosity in the r-, φ- and z-directions of a polar system. Based on that, predictions of the bulk temperature with an average error <5% were obtained with the reference point being defined as the core of the material string ejected to the atmosphere [12]. Fasching et al. [14,15] showed in subsequent research the importance of correct determination of the mass temperature profile after dosing. Direct measurement of this temperature in the plasticizing cylinder is technically not possible, which is why the initial mass temperature in simulations often equals the set (constant) temperature of the plasticizing cylinder defined at the injection molding machine [16]. Therefore, Fasching proposed a higher initial set temperature for the rubber compound applied in simulation. Consequently, he succeeded in reducing the deviation between simulated temperature curves of an injection molding process and the actual temperature of the rubber compound during processing. Pioneering work for the determination of the transient mass temperature profile during the dosing process was carried out by Kerschbaumer et al. [17,18]. Using an engineering approach, they designed a test stand consisting of a plasticizing cylinder equipped with pressure sensors, a throttle valve for back pressure adjustment, and a measurement bar containing thermocouples for the determination of the actual state of the mass temperature. Based on this new method, the group provided evidence of the well-known dependency of the compound temperature on the settings of the dosing step [9], which is attributed to the screw rotational speed and the back pressure.

The present work aimed at combining the results of the above mentioned research of Perko et al. [12,13], Fasching et al. [14,15], and Kerschbaumer et al. [17,18], and intends to reveal the advantage of considering the actual state of the mass temperature profile in process simulations, which consequently improves virtually obtained results.The target value for the comparison of the simulated and the real processes is defined in this work as the temperature measured in the mold during injection. In addition, the numerically calculated relative degree of cure was contrasted with the mechanically determined reaction progress by means of a compression set (CS) and dynamic-mechanical analysis (DMA) in order to demonstrate the effect of considering the temperature profile in injection molding simulations.

## 2. Materials and Methods

### 2.1. Material and Equipment

All experiments were carried out with styrene butadiene rubber (SBR) supplied from Semperit Technische Produkte GmbH (Wimpassing, Austria). The compound is filled with carbon black and uses a sulfur-based curing system and other additives. However, the exact quantities remain confidential, since the compound is currently used for industrial purposes. Material characterization for the process simulation in terms of reaction kinetics and viscosity were conducted on a dynamic rubber process analyzer (D-RPA) 3000 from MonTech Werkstoffprüfmaschinen GmbH (Buchen, Germany) and a high pressure capillary rheometer (HPCR) of the type Rheograph 50 from GÖTTFERT Werkstoff-Prüfmaschinen GmbH (Buchen, Germany). Thermal conductivity and specific heat capacity of the rubber compound were measured using a guarded heat flow meter (GHF) DTC 300 from TA Instruments (New Castle, DE, USA) and a differential scanning calorimeter from Mettler-Toledo International Inc. (Greifensee, Switzerland), respectively. Measurements of the specific volume as a function of pressure and temperature (pressure volume temperature, PVT) were conducted on the PVT-100 from SWO Poylmertechnik GmbH (Krefeld, Germany).

### 2.2. Experimental Setup

#### 2.2.1. Injection Molding Machine

Experiments for the verification of the correlation of a real rubber injection molding process with simulation of the same were conducted on a rubber injection molding machine MTF750/160edition from Maplan GmbH (Kottingbrunn, Austria). The machine was equipped with a 4-cavities-mold, including a cold runner system from PETA Formenbau GmbH (Bad Soden-Salmuenster, Germany). By means of pressure and temperature (pT)-sensors from Kistler Instrumente AG (Winterthur, Switzerland) placed close to the inlet in the cold runner section of the mold and in one of the cavities, the mass temperature was obtained continuously during a process cycle. The acquired data were processed and employed for the comparison of the real process and the simulation. Machine settings of the conducted experiments are shown in Table 1. The shot volume for complete filling of the cavities was 208 cm^3^ throughout all experiments. The cure time was varied and started at the lowest possible cure time to generate just enough stability to eject the parts to fully cured parts.

#### 2.2.2. Test Stand

**Figure 1:**
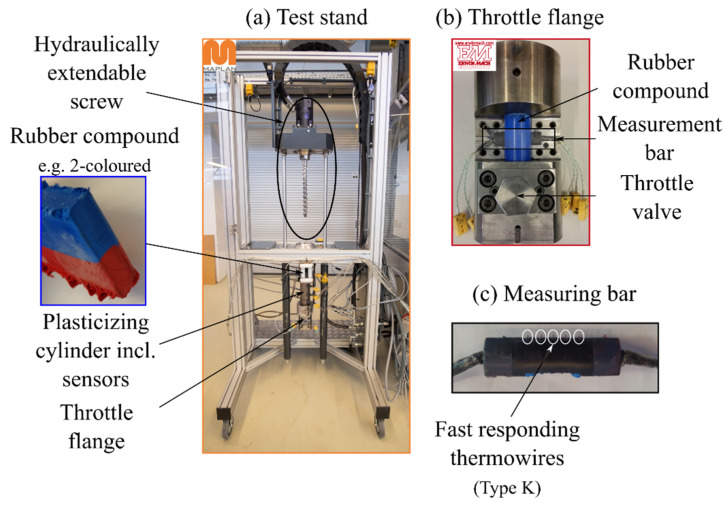
Test stand used for the process-orientated determination of the axial mass temperature profile during dosing: (**a**) test stand consisting of a “First-In-First-Out” dosing unit with sensors and equipped with a (**b**) throttle flange that contains the (**c**) measuring bar [18].

The test stand designed by Kerschbaumer et al. [17] shown in Figure 1 was employed in this work for the determination of the axial temperature profile of the rubber compound during dosing. Relevant process settings are listed in Table 1. The core part of this unique setup consists of a dosing unit of the “First-In-First-Out” (FIFO) rubber injection molding machine MTF750/160edition manufactured by MAPLAN GmbH (Kottingbrunn, Austria) with a hydraulically removable screw. In addition, the unit is equipped with a throttle flange with the backpressure control option from EM Gummitechnik (Hirm, Austria), to which the measuring bar with fast responding thermocouples can also be mounted. Furthermore, a pressure sensor located in the plasticizing cylinder is used to verify the conditions during the experiments.

The measuring process of the temperature profile with the test stand first requires the pressure conditions in the screw antechamber to be adjusted. Unlike conventional injection molding machines, which control the pressure themselves, this is not possible with the test stand as the compound is dosed into the atmosphere. The pressure setting at a desired screw rotational speed is therefore achieved with the throttle valve. Since the test stand can only dose a mass for a defined time instead of a defined volume, the required dosing time for a defined volume must be determined gravimetrically in the next step. This is possible due to a linear correlation of dosing volume and time. As soon as these two criteria are met, the temperature profile for a desired screw rotational speed and back pressure can be determined [17].

#### 2.2.3. Injection Mold and Rubber Part

**Figure 2:**
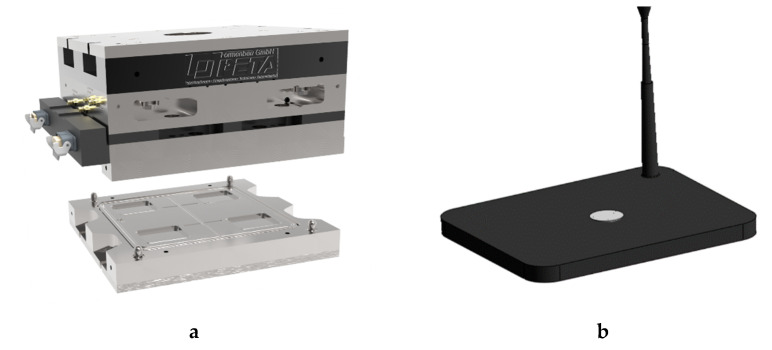
(**a**) 4-cavities-mold with a cold runner system by PETA Formenbau GmbH used for manufacturing injection-molded rubber parts; (**b**) mechanical testing of the curatives conducted by means of dynamic-mechanical analysis (DMA) and compression set analysis with samples taken from the marked position.

The verification of the virtually obtained results was performed on an injection mold designed by PETA Formenbau GmbH (Bad Soden-Salmuenster, Germany). After the compound is dosed in the screw antechamber, the material is injected into the mold, first entering the cold runner section with a symmetrically arranged distributing system, and then passing through conical dies to fill the four heated cavities where the kinetic reaction occurs. The process settings used in the experiments are listed in Table 1. Both, the 4-cavities-mold and the shape of an injection-molded rubber part are depicted in Figure 2a, b. The DMA and compression set analysis methods for determining the mechanical behavior of the vulcanized rubber parts are discussed in detail in a later section.

#### 2.2.4. Simulation

The numerical calculations of the injection molding processes conducted in practical experiments on the machine were done with the 5.3.0.6 release of SIGMASOFT^®^ from SIGMA Engineering GmbH (Aachen, Germany). Three basic approaches for defining the inlet temperature were chosen in the simulation to obtain the desired comparison of mass temperature profiles in the mold for both the virtual and the real processing:

**1.**  Simulation of the process cycle assuming that the inlet temperature of the compound equals the set temperature of the dosing unit.
**2.**  Simulation of the process cycle assuming that the inlet temperature of the compound is higher than the set temperatures in the dosing unit, but still constant, thus corresponding to Fasching’s hypothesis [14].
**3.**  Simulation of the process cycle considering the actual state of the mass temperature profile at the test stand for the given settings in Table 1.

**Figure 3:**
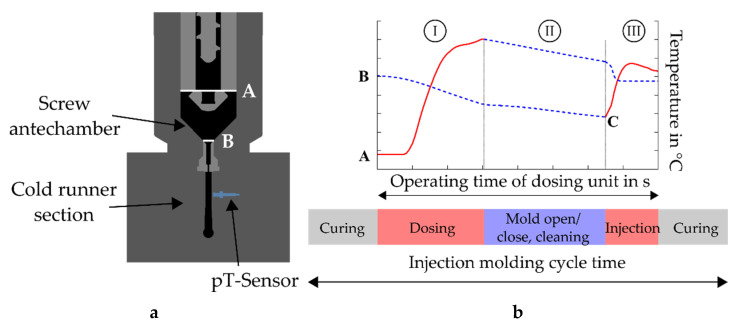
(**a**) Simplified simulation setup considering the measured temperature profile at inlet A to calculate the profile during injection at point B; (**b**) simulated temperature profiles during the operating time of the dosing unit, where C indicates the set temperature for simulations without the dosing unit. The three sections describe the phases (I) dosing, (II) retention time, and (III) injection of the entire injection molding process.

The most important question in the preparation of any simulation is how detailed the program should be and which parts of a mold, i.e., plates with runners, cooling systems or screws, are considered. Consequently, the parameters for the mesh formation are defined, however, taking into account that finer settings will extend the required time for the simulation run. In addition, the time is also extended if the software is to consider the heating process. This is particularly important for those simulations where the measured temperature profile is taken into account, which is why they were divided into two runs in this work as follows:

In the first run, the simulation aimed to determine the temperature profile occurring during injection of the compound. This run assumes a constant temperature of the dosing unit and cold runner sections; thus, heating is not taken into account. Figure 3a illustrates the setup for a simplified simulation. The compound is dosed through an inlet A, considering the obtained temperature profile from the test stand experiments, until the screw antechamber between A and B is filled. Next, a retention time is regarded covering the steps of mold opening/closing, part ejection, and cavity cleaning of a real injection molding process. Finally, the metered compound is injected at a certain velocity through B and the cold runner section. In Figure 3b an illustrative temperature profile is shown for the three sections (I) dosing, (II) retention time, and (III) injection. The solid lines in sections I and III indicate the measured temperature profile during dosing and the simulated mass temperature obtained in the injection phase, respectively. The dashed lines show a loss of heat, as there is no movement for a certain period.

The second simulation run then calculates the desired injection molding process, whereby the inlet is at B (see Figure 3b) and the dosing unit is neglected. Hence, the temperature profile for the injection of the rubber compound calculated in the previous run is applied starting from C (Figure 3b). Moreover, the mold-heating phase is considered in this run.

All other simulations executed in this work, which consider a constant set temperature of the compound at the inlet rather than a temperature profile, were conducted in one run, taking the heating phase into account. General settings of the injection molding simulation are listed in Table 2.

#### 2.2.5. Characterization

**Figure 4:**
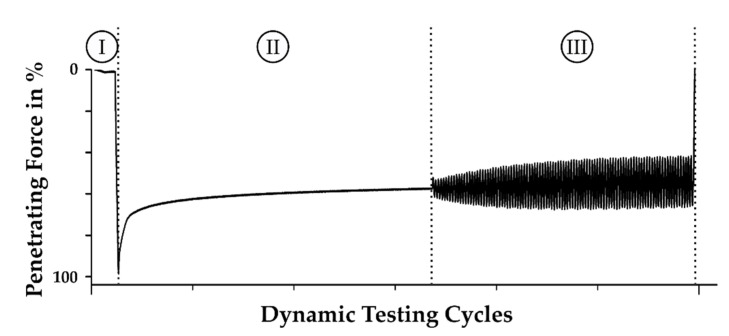
Test sequence for the DMA measurements, starting with a sequence for (I) maximum penetration, followed by a sequence of (II) relaxation and (III) cycles of dynamical testing [20,21].

The verification of the experiments conducted at the injection molding machine and with the simulation was not only reduced to temperatures measured during the cycle, but also based on the mechanical testing of the rubber parts obtained. Compression set analysis was done following the standards described in DIN ISO 815-1 [19]. Samples with dimensions of 13 mm diameter and 6.3 mm height were taken from the middle region of the injection-molded parts and tested accordingly. In addition, DMA studies were performed using an ElectroPulsTM E3000 All-Electric Dynamic Test Instrument from Instron GmbH (Norwood, MA, USA) with the test sequence shown in Figure 4. The measurement is based on a force-controlled test sequence including the determination of the maximum depth of penetration, followed by relaxation, and completed by 100 cycles of dynamical testing [20,21]. The latter is derived as hysteresis in the material data set and is transformed into the dynamic spring constant *C*_dyn_, following:

(1) $$C_{\mathrm{dyn}}=\frac{\Delta F}{\Delta x}$$

where Δ*F* and Δ*x* represent the delta of the force and the deformation path, respectively.

## 3. Results and Discussion

**Figure 5:**
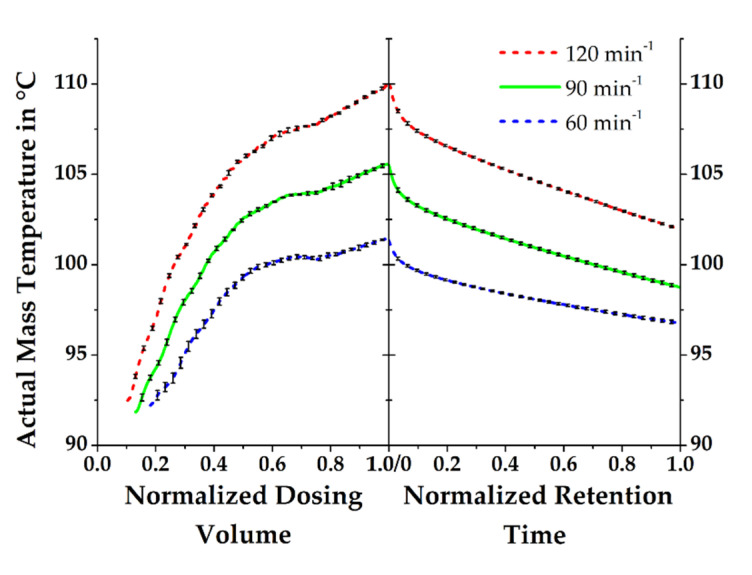
Actual mass temperatures obtained at screw rotational speeds of 60, 90, and 120 min^−1^. The rubber compound gains heat due to dissipation during dosing and emits heat during the retention time.

The common practice of setting up an injection molding simulation revealed that the initial mass temperature is often assumed to equal the set temperature of the plasticizing cylinder of an injection molding machine. Ramorino et al. [16] recently compared results from a real manufacturing process and simulation using an acrylonitrile butadiene rubber (NBR) and they hypothesized that the initial mass temperature had negligible effects on the results of the numerical calculations. Contrary findings were obtained in this work by measuring the actual state of the mass temperature profile with the previously described test stand. An illustration of the result is given in Figure 5, which shows the relevant temperature profile for the present work at a screw rotational speed of 90 min*^−^*^1^. As a result of the movement of the rubber compound through the plasticizing cylinder induced by the screw rotation and the simultaneous heat dissipation, the material continuously gains heat until dosing is finished at a defined volume. The amount of heat gained by the system is obviously related to the screw rotational speed, which is underlined by the depiction of additional temperature profiles measured at 60 and 120 min*^−^*^1^. Consequently, the significance of the actual mass temperature after dosing for the purposes of reliable process simulation is made evident.

**Figure 6:**
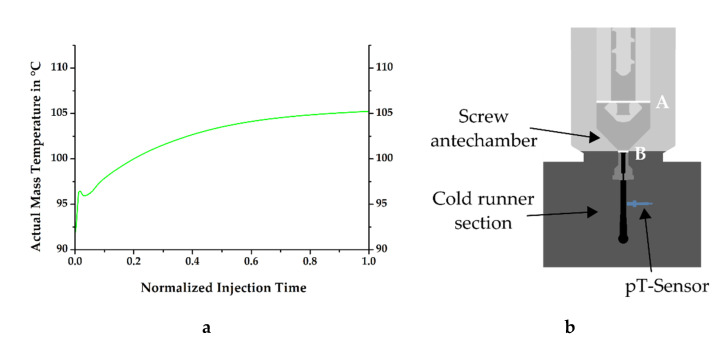
(**a**) Numerically determined mass temperature obtained at plane B after preliminary dosing with a screw rotational speed of 90 min*^−^*^1^ and with an injection speed of 18 cm^3^ s^−1^; (**b**) Simplified simulation setup showing the position of the appliance for the calculated mass temperature profile during injection at B.

At the injection molding machine, the process is defined such that a retention time follows the dosing step, during which no material movement is seen, but heat transfer is observed from the rubber compound to the dosing unit. This reduction of temperature in the material must be quantified for process simulation, as it defines the initial mass temperature at the inlet of the mold. However, a direct measurement of this temperature is not feasible due to the technical design of the test stand and can only be calculated. Therefore, the determination was carried out with the help of simulation software, resulting in a temperature curve as shown in Figure 6a, which indicates the thermal conditions during injection at the previously described plane B (see Figure 6b). Even though the temperature of the dosing unit was set to 80 °C at this position, it can be clearly seen that the mass temperature was considerably higher, confirming the hypothesis of Fasching concerning the simulation definitions of the considered material at the inlet [14].

**Figure 7:**
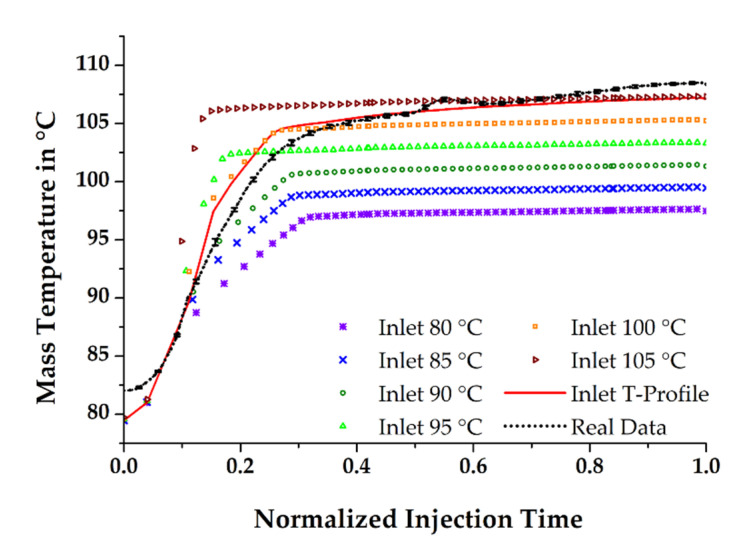
Mass temperature obtained at the surface area of the pT-sensor after various definitions of the compound’s inlet temperature and opposed to the measured curve from the injection molding process.

Based on these findings, it becomes evident that the consideration of a temperature profile has significant effect on the further thermal history of the rubber compound in the mold, regardless of which injection molding process is to be validated numerically. Moreover, the simulations conducted in this work have shown that it is also not sufficient to define an inlet temperature of the material that is higher than the set temperature of the dosing unit. Figure 7 compares the mass temperatures obtained in the simulations during filling with the real process data at the surface area of the pT-sensor in the cold runner. The purple curve with star-like marks in the graph, resulting from the simulation run, where the inlet temperature of the mass equals the nominal temperature of the dosing unit, shows once more that the further thermal history of the material in the given situation is far from being comparable with the real data. Apparently, this is the result of neglecting the shear-induced temperature increase in the dosing phase. This difference has significant effects on the state of cure in the molded part, as will be shown later.

For the subsequent process simulations, the starting temperature at the inlet was increased to 105 °C in 5 K steps. The results show a stepwise approach of the simulated temperature to the real measurements. Nevertheless, none of the calculated runs enabled an entire match between simulation and process data. On the one hand, the curve contributing to an initial mass temperature of 100 °C fits well in the first phase of the filling process, where the system moves towards a constant mass flow, but slightly deviates in the remaining filling time. On the other hand, the plateau region of the temperature curves, which is associated with continuous flow conditions, is approximately consistent with the real data when the material enters the mold with a constant temperature of 105 °C. In this case, however, the initial temperature rise significantly mismatches the measured values. The only run with satisfactory compliance in both phases of the filling process was obtained when the axial mass temperature profile during filling, determined from a previous run and derived from the experiments at the test stand, was included.

**Figure 8:**
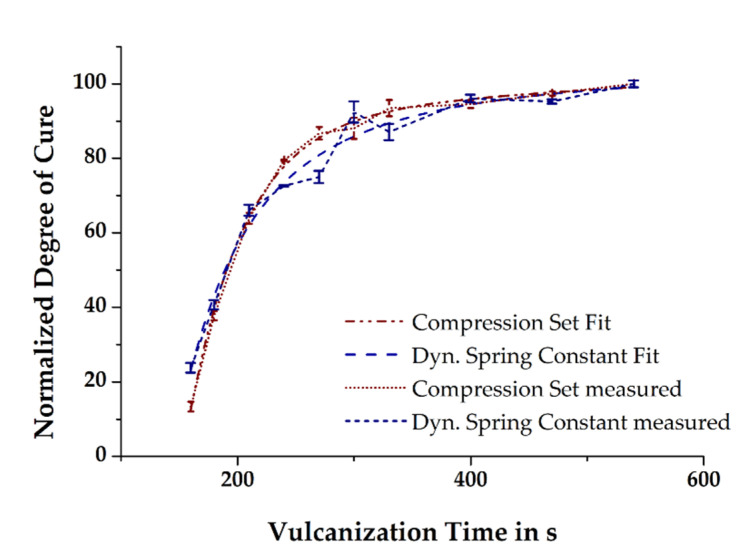
Data fit of normalized relative degree of cure calculated from the compression set (CS) and DMA measurements, respectively.

In the previous section, it was briefly mentioned that the simulated state of cure in the molded part depends on the initial mass temperature, among other factors. Thus, it is hypothesized that the more accurate is the prediction of the relative degree of cure in the simulation compared to that of the measured relative degree of cure in injection-molded rubber parts, the better is the temperature profiles of the simulated and the real process match. The relative degree of cure of the moldings vulcanized at 160 °C and in the time range 160–540 s was determined by means of CS and the dynamic spring constant was derived from DMA. Assuming the vulcanization reaction to be completed within the given maximum time, the results obtained were normalized by taking into account the time dependency. Ultimately, curve fitting of the normalized data demonstrates the excellent comparability of the two different analytical methods (see Figure 8) and thus provides the opportunity for a direct correlation of the measured mechanical properties with the simulated relative degree of cure.

**Figure 9:**
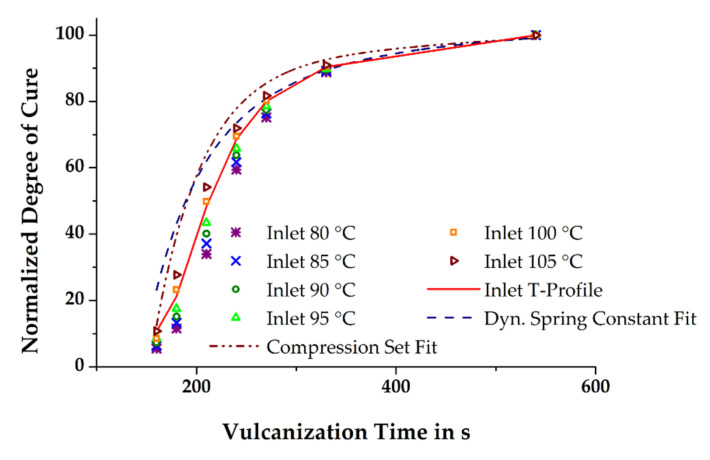
Comparison of the normalized relative degree of cure obtained from simulation and mechanical testing of injection-molded rubber parts.

The corresponding progress of the vulcanization reaction in the simulation was calculated with the SIGMASOFT^®^-specific function “Evaluation Area”, which determines the mean values of a desired part, e.g., the relative degree of cure, at a preliminary defined time and volume. For this work, the volume was the size of a CS-sample. Figure 9 depicts the calculated normalized degree of cure of the conducted numerical experiments and compares simulation and mechanical characterization. Firstly, it is evident that the reliability of the numerically-determined results is significantly improved when the thermal history of a rubber compound related to the dosing phase is considered in injection molding simulation. Significant deviation of the relative degree of cure is obtained if the initial set temperature of the rubber compound equals the definitions in the dosing unit of the machine. Due to the missing inclusion of the additional, shear-induced heating of the rubber compound during the dosing phase, the progress of the vulcanization reaction is substantially slower. By increasing the temperature of the material at the entry in steps of 5 K, thus approaching the actual shear-affected mass temperature in steps, the numerically-determined relative degree of cure and the measured results consequently converge. Similar to the previously shown results of the mass temperature passing the sensor point in the cold runner section, the best coherence of the relative degree of cure for a simulation run with a constant inlet temperature of the mass with the run, considering the measured axial mass temperature profile, is obtained at a set temperature of 100 °C. However, this is not simultaneously valid for the comparison of simulation and real process data. Interestingly, the best match was observed with the simulation having a constant initial mass temperature of 105 °C, especially at short vulcanization times, even though the virtual process with the considered axial mass temperature profile is expected to fit best. A conclusive explanation for this is not readily apparent.

## 4. Conclusions

Shear heating is a physical process in rubber injection molding that begins before the entry of the compound into the mold at the first moment after passing the feed and continues in the transport through the dosing cylinder. Neglecting this effect in process simulations inevitably leads to significant deviations of the mass temperature in the cold runner, and ends in a potentially considerable difference of the vulcanization reaction. The most accurate results in the comparison of real process and simulation were observed when applying the axial mass temperature profile measured with the test stand in the numerical calculations. Due to the consideration of the compound’s thermal history before entering the mold, a very good reproducibility of the temperature curve was obtained. Furthermore, the relative degree of cure calculated from mechanical testing methods and observed in the process simulation revealed a substantially higher compliance when considering the axial mass temperature profile after dosing.

## Figures and Tables

**Table 1:** Process settings for the experimental work. Here, ^a^ indicates the parameters relevant for the test stand while ^b^ represents settings relevant to the injection molding machine.

Temperature Dosing Unit ^a,b^ (°C)	Temperature Cold Runner ^b^ (°C)	Temperature Cavities ^b^ (°C)	Screw Rotational Speed ^a,b^ (min^−1^)	Injection Speed ^b^ (cm^3^ s^−1^)
80	80	160	90	18

**Table 2:** Simulation settings of the injection molding process; Here, ^a^ indicates that the screw rotational speed has to be considered in those simulation runs only where a measured profile of the mass temperature was applied. Thermal conductivity: steel λ_S_ = 39 Wm^−1^K^−1^, heat transfer coefficient: steel-steel 10,000 Wm^−2^K^−1^, steel-rubber 800 Wm^−2^K^−1^.

Temperature Cold Runner (°C)	Temperature Cavities (°C)	Screw Rotational Speed ^a^ (min^−1^)	Injection Speed (cm^3^ s^−1^)	Curing Time Range (s)
80	160	Measured Temperature Profile	18	160–540

## Data Availability

The data presented in this study are available upon request from the corresponding author and after consultation with our partners. The data are not publicly available as they are part of an ongoing study.
